# Assessment of microfungal contamination and enzymatic activity in ethnographic textile artifacts and museum environment

**DOI:** 10.1371/journal.pone.0339319

**Published:** 2026-01-14

**Authors:** Guven Ozdemir, Alev Haliki, Melike Çelebi

**Affiliations:** Department of Biology, Section of Basic and Industrial, Microbiology, Ege University, Izmir, Turkey; Satyawati College, University of Delhi, INDIA

## Abstract

Ethnographic textile artifacts are highly susceptible to fungal biodeterioration due to their organic composition and continuous exposure to microfungi in museum environments. This study aimed to assess the extent of microfungal contamination in the exhibition and storage areas of the Ege University Ethnography Museum and to evaluate the enzymatic activities (cellulase and protease) of the isolated fungal species to determine their biodeterioration potential. Air and surface samples were collected from display halls, storage rooms, and outdoor reference points during two seasons (spring and autumn) using a portable air sampler on DG-18, PCA, and MEA media. Fungal isolates were identified through macroscopic and microscopic examination supported by standard mycological keys, and their enzymatic activity was assessed using CMC agar for cellulase and skim milk agar for protease production. Microclimatic influences and seasonal differences were statistically evaluated using a two-sample independent t-test. Fungal load ranged from 120–450 CFU/m³ on DG-18 and 300–1000 CFU/m³ on PCA, with the highest values recorded inside display cases and storage zones. A total of 58 fungal isolates were obtained, predominantly belonging to *Aspergillus, Penicillium*, *Cladosporium, Alternaria*, and *Rhizopus*. Enzymatic assays showed that several isolates exhibited strong cellulase and protease activities, particularly *A. sydowii, P. citrinum, A. flavus*, and *P. chrysogenum*, indicating a high biodeterioration risk for cellulose- and protein-based textiles. Seasonal differences were statistically insignificant, highlighting the greater importance of microclimatic conditions and ventilation patterns. These findings underscore the need for integrated biological risk management and continuous microbial monitoring to protect ethnographic textile heritage from fungal deterioration.

## 1. Introduction

Ethnographic textile artifacts are an important part of cultural heritage, serving as a bridge between past and present. However, these organic materials are vulnerable not only to physical and chemical factors but also to biological agents in the museum environment. In particular, microfungi (molds and mildew) can cause severe biodeterioration of such materials due to their strong enzymatic activities [1,2]. Microscopic fungi possess a rich enzymatic repertoire capable of degrading resistant biopolymers such as cellulose, lignin, and keratin, making them primary decomposers in nature [1]. Consequently, fungi are among the most destructive agents affecting historical artifacts in both indoor and outdoor settings, causing physical damage, chemical degradation, and aesthetic deterioration (e.g., staining, discoloration) [3].

At a broader scale, recent reviews have synthesized current knowledge on fungal biodeterioration and preservation of cultural heritage. One comprehensive review highlighted how filamentous fungi colonizing heritage materials can combine pigment and organic acid production with extensive extracellular enzyme activity to drive both physical and chemical degradation under a range of environmental conditions [4]. These works collectively underscore the need for integrated approaches that link fungal diversity, airborne and surface contamination levels, and enzymatic activity to site-specific microclimatic parameters. However, there is still a paucity of studies that simultaneously quantify airborne fungal load, identify microfungi associated with ethnographic textile artifacts, and evaluate their cellulolytic and proteolytic activities within a single museum context, particularly in the Eastern Mediterranean region.

The natural fibers found in textile artifacts—such as cellulosic fibers (cotton, linen) and protein-based fibers (wool, silk)—serve as nutrient sources for microfungi and can be rapidly colonized under humid conditions. During their growth, microfungi secrete hydrolytic enzymes such as cellulases and proteases, which degrade the organic material and compromise the structural integrity of the artifacts [4,5]. Additionally, the production of organic acids and pigments by these fungi can lead to irreversible staining and color changes in textiles [2]. If left uncontrolled, microfungal biodeterioration can severely damage the fibrous structure of historical textiles, resulting in loss of durability and permanent harm.

Therefore, microbiological risk management is crucial for the preservation of museum artifacts. In ethnographic museums, textile items such as garments, carpets, rugs, and covers are continuously exposed to fungal spores due to dust accumulation and visitor traffic. Without proper temperature and relative humidity control, these spores can settle and actively proliferate on the artifacts. Once colonized, microfungi pose a threat not only to the affected item but also to surrounding objects. Museum conservation units must implement preventive strategies such as maintaining controlled temperature and humidity, reducing biological load through regular cleaning, monitoring air quality, and conducting periodic microbial assessments of artifact surfaces [6].

Recent reviews have underscored the increasing risk posed by microfungi to textile cultural heritage. One review emphasized that fungi represent one of the most destructive agents affecting historical textiles due to their ability to secrete cellulases, proteases and other hydrolytic enzymes that progressively weaken natural fibers. This review also highlighted a major limitation in the field: although the biodeterioration pathways of textiles are well documented, relatively few studies provide quantitative microbial load data or directly relate enzymatic activity to museum-specific microclimatic conditions, creating a gap that remains to be addressed [7].

Several previous studies have demonstrated that dominant microfungal communities vary depending on the type of artifact. For instance, one study reported high prevalence of *Aspergillus niger*, *Penicillium chrysogenum*, and *Alternaria alternata* on historical textiles in Egypt [8,9]. Similarly, another study investigated fungal colonization on a historical mummy and found that the isolates exhibited high cellulase, chitinase, and protease activities. These findings highlight the importance of evaluating not only the presence of fungal colonies but also the enzymatic profiles of the species to assess their potential for artifact damage [10].

More recent experimental studies have provided additional insights into fungal colonization of historical textiles. One study examined microfungi isolated from ancient wool and linen artifacts and demonstrated their ability to induce both structural and color changes through pigment production and metabolic activity. Nonetheless, their study was limited to a small number of artifacts and did not include an evaluation of indoor air quality or a systematic analysis of fungal enzymatic profiles, highlighting the need for more integrated approaches [11].

Fungal pigment production is another critical factor contributing to the visual deterioration of artifacts. Pigments derived from fungal metabolites, particularly melanin-based compounds, can cause staining, discoloration, and aesthetic degradation of textiles [2]. Species such as *Aspergillus fumigatus* and *A. sydowii* are known to produce dark green or blackish pigments in culture, which can lead to permanent color alterations in fabrics.

Moreover, climatic conditions play a significant role in the deposition and colonization of fungal spores on artifact surfaces. Previous studies have shown that fungal spore concentrations in museum indoor air tend to increase during warmer seasons. Other research has observed temporal and spatial variations in fungal diversity and abundance at cultural heritage sites, emphasizing the influence of seasonal changes on microbial load. Additional investigations have detected high levels of fungal contamination in the indoor air of historical museums and evaluated the impact of ventilation systems on microbial burden. These findings underscore the importance of seasonal optimization of museum climate control systems and continuous air quality monitoring [12,13].

Complementing these earlier findings, a recent study assessed airborne and surface-associated microorganisms in several museums located in arid regions of China and reported notable spatial variation in CFU/m³ values depending on ventilation and humidity levels. However, the study primarily focused on overall microbial burden and did not investigate the enzymatic deterioration potential of the fungi, leaving an important aspect of biodeterioration unaddressed [14].

Nanotechnological approaches are also gaining importance in conservation strategies. The application of biopolymer-based materials such as chitosan to textile surfaces has shown promising antifungal and antibacterial effects, contributing to the protection of artifacts against biological deterioration. Recent research reported reported successful outcomes using chitosan modification technology to reduce microbial contamination on textile surfaces. Such treatments are considered invisible and non-toxic protective agents suitable for museum environments [15].

Biological risk assessment in cultural heritage conservation should not be limited to identifying destructive organisms; it must also consider their enzymatic capabilities, spore dispersal potential, and environmental adaptability. The literature emphasizes that effective conservation strategies require a holistic evaluation of these factors [4].

In addition to microbiological analyses, advanced imaging and eco-friendly conservation technologies have also been explored. An interdisciplinary framework combining digital imaging techniques with natural compound–based eco-cleaning agents has been proposed to enhance the preservation of textile cultural heritage. While their approach demonstrated promising results for non-invasive documentation and surface cleaning, it did not address airborne fungal contamination or the enzymatic biodegradation potential of microfungi—two aspects that are central to understanding biodeterioration risks in museum environments [16].

This study aims to identify microfungi present on ethnographic textile artifacts and in the indoor air of the Ege University Ethnography Museum, and to evaluate their enzymatic activities (cellulase and protease production potential). By doing so, the microbiological risk level of the museum environment will be revealed, contributing to the development of scientifically grounded strategies for the preservation of cultural heritage textiles.

## 2. Materials and methods

### 2.1. Study area

**Fig 1:**
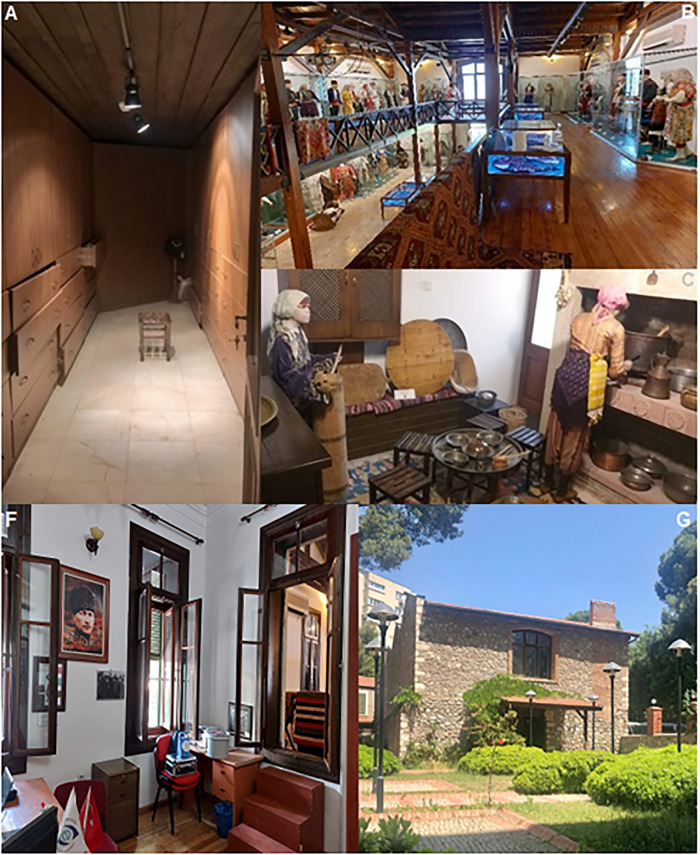
Representative photographs of sampling locations in the Ege University Ethnography Museum. **(A)** Storage corridor used for artifact preservation (lower floor), **(B)** Upper floor exhibition hall containing ethnographic textiles and costumes **(C)** Ethnographic kitchen display (lower floor), **(D)** Traditional garment display – Kosovo/Prizren section (upper floor), **(E)** Zeybek traditional costume showcase representing an IDC sampling point, **(F)** Staff office room sampled as part of indoor environmental monitoring, **(G)** Outdoor museum environment representing the OME sampling point.

This study was conducted at the Ethnography Museum of Ege University, located in İzmir, Türkiye. The museum is located in Bornova (İzmir, Türkiye) at coordinates 38.460998° N, 27.217161° E, approximately 6 km inland from the Aegean coastline. The investigation focused on traditional textile artifacts in the museum’s collection (such as garments, covers, carpets, etc.) as well as textile materials stored in the depot area. Microbiological sampling was performed during two different seasons (spring and autumn), taking into account the microclimatic variability within the museum. Sampling points were selected based on parameters such as visitor traffic, humidity intensity, and artifact density. All sampling procedures were carried out with official permission granted by the Ege University Ethnography Museum Directorate. This study did not involve human or animal subjects, and therefore ethics committee approval was not required. All samples were limited to environmental air and artifact surface assessments and were collected solely under institutional permission.Representative photographs of the sampling environments, including the kitchen exhibit, storage corridor, exhibition halls, staff office, and outdoor museum surroundings, are provided in Fig 1 to illustrate the spatial context and variety of sampled locations.

### 2.2 Sampling method

To determine the fungal and bacterial load in the museum’s indoor air, a portable air sampler (Merck MAS-100) was used. Air samples were collected from a total of 22 different locations. During sampling, DG-18 (Dichloran Glycerol 18%) and PCA (Plate Count Agar) media were used. DG-18 was selected to assess fungal contamination, while PCA was used to evaluate the total aerobic microbial load. Sampling was conducted at 14 display case locations (IDC), 7 non-display museum interior locations (ODC), and 1 outdoor museum environment location (OME). Each sampling point was analyzed in triplicate.

### 2.3. Incubation and colony counting

Petri dishes were incubated in an inverted position at 25–30°C for 5–7 days. Developed colonies were counted macroscopically, categorized by colony type, and the fungal concentration in air samples was calculated in CFU/m³ using the following formula:

CFU/m³ = (Total Colony Count × 1000)/Sampled Air Volume (L)

### 2.4. Isolation and ıdentification of fungal ısolates

Colonies with distinct morphologies were purified by spot inoculation onto Potato Dextrose Agar (PDA) to obtain pure cultures. The isolates were identified based on macroscopic characteristics (colony color, texture, surface structure, pigment production) and microscopic features (spore structure, hyphal morphology). Identification was supported by standard mycological keys [17,18].

### 2.5. Enzymatic activity assays

#### 2.5.1. Cellulase activity.

To determine the cellulolytic potential of the isolates, Carboxymethylcellulose (CMC) agar medium was prepared with the following composition: 1% CMC, 0.2% NaNO₃, 0.1% KH₂PO₄, 0.05% MgSO₄·7H₂O, 0.05% KCl, and 2% agar. The medium was sterilized and poured into Petri dishes. Each isolate was inoculated by spot placement at the center of the plate. After incubation at 28°C for 5 days, the plates were stained with 0.1% Congo red for 20 minutes and then rinsed with 1M NaCl to visualize the cellulose hydrolysis zones [19]. Enzymatic activity was evaluated by calculating the ratio of the hydrolysis zone diameter to the colony diameter.

#### 2.5.2. Protease activity.

For proteolytic activity, 10% Skim Milk Agar was prepared. Isolates were inoculated in the same manner and incubated at 28°C for 5 days. The formation of clear zones in the opaque medium indicated protease activity, which was similarly assessed by the ratio of the zone diameter to the colony diameter.

### 2.6. Statistical analysis

To evaluate seasonal differences in microbial load, a two-sample independent t-test was performed comparing CFU/m³ values obtained in September and May across all sampling locations and media types (DG18 and PCA). The test was conducted using a significance level of 0.05 to determine whether seasonal variation had a statistically significant effect on microbial concentrations.

### 2.7. Methodology workflow

**Fig 2:**
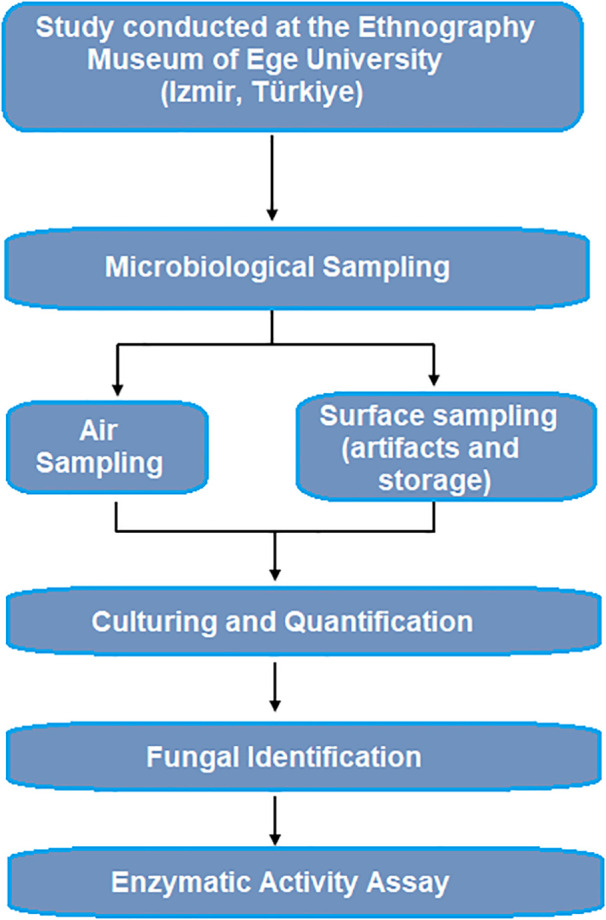
Workflow diagram summarizing the methodological steps of the study.

To provide a clear overview of the methodological steps applied in this study, a workflow diagram summarizing the entire process—from environmental sampling to fungal isolation, identification, and enzymatic activity assessment—is presented in Fig 2. This diagram illustrates the sequential stages of air and surface sampling, culture-based enumeration, purification of isolates, morphological identification, and cellulase/protease activity testing, as well as the statistical evaluation of seasonal variation.

All relevant data supporting the findings of this study are openly accessible in Zenodo under the DOI: https://doi.org/10.5281/zenodo.17863876. The dataset includes airborne CFU measurements and fungal load values obtained from indoor and outdoor sampling locations across two seasons.

## 3. Results

Microbiological sampling conducted at the Ege University Ethnography Museum revealed both quantitative and qualitative aspects of biological risk within the museum environment. Air sampling results showed that, in September, fungal concentrations in DG-18 medium ranged from 95 to 605 CFU/m³ inside display cases, while total aerobic microbial load in PCA medium ranged from 0 to 420 CFU/m³. In May, DG-18 values ranged from 150 to 590 CFU/m³, and PCA values from 0 to 645 CFU/m³.

Outside the display cases, fungal concentrations in September ranged from 125 to 1400 CFU/m³ in DG-18, and from 290 to 1470 CFU/m³ in PCA. In May, DG-18 values ranged from 320 to 1090 CFU/m³, and PCA values from 150 to 2040 CFU/m³.

**Fig 3:**
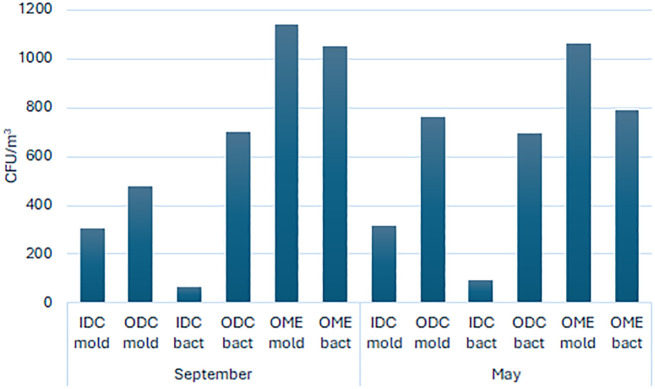
Distribution of average microbial load (CFU/m³) measured in DG-18 (fungal) and PCA (bact., bacterial)) media across different sampling locations, including inside display cases (IDC), outside display cases (ODC), and outdoor museum environment (OME), during September and May sampling periods.

The highest contamination levels were observed inside display cases and in storage areas, which were associated with microclimatic factors such as low air circulation and high humidity. Fig 3 illustrates the distribution of average microbial load (CFU/m³) across sampling points in DG-18 and PCA media. The graph highlights regional differences in indoor air quality, indicating that poorly ventilated areas such as storage zones and display interiors pose a higher biological risk.

The results of the independent t-test revealed no statistically significant difference in microbial load between September and May samples (**t = 0.0175**, **p = 0.9864**). This suggests that seasonal variation did not significantly affect the overall CFU/m³ levels in the museum environment. Therefore, other factors such as sampling location and microclimatic conditions may play a more dominant role in microbial distribution.

A total of 58 fungal isolates were obtained, of which 44 were phenotypically identified at the species level. Table 1 shows the species isolated from IDC (inside display cases), ODC (outside display cases), and OME (outdoor museum environment).

**Table 1:** Identified fungal species isolated from IDC, ODC, and OME.

IDC	ODC	OME
*Alternaria alternata*	*Aspergillus aculeatus*	*Aspergillus candidus*
*Alternaria longipes*	*Aspergillus alliaceus*	*Aspergillus carbonorius*
*Alternaria sonchi*	*Aspergillus candidus*	*Aspergillus flavus*
*Aspergillus aculeatus*	*Aspergillus flavus*	*Aspergillus kanagawaensis*
*Aspergillus amstelodami*	*Aspergillus insuetus*	*Aspergillus niger*
*Aspergillus candidus*	*Aspergillus japonicus*	*Penicillium decumbens*
*Aspergillus cervirus*	*Aspergillus melleus*	*Penicillium janczewskii*
*Aspergillus flavus*	*Aspergillus niger*	
*Aspergillus japonicus*	*Aspergillus parasiticus*	
*Aspergillus kanagawaensis*	*Aspergillus penicillioides*	
*Aspergillus melleus*	*Aspergillus wentii*	
*Aspergillus niger*	*Cladosporium cladosporioides*	
*Aspergillus niveus*	*Cladosporium sp.*	
*Aspergillus ochraceus*	*Fusarium avenaceum*	
*Aspergillus ostianus*	*Penicillium citreonigrum*	
*Aspergillus parasiticus*	*Penicillium citrinum*	
*Aspergillus sydowii*	*Penicillium decumbens*	
*Aspergillus terreus*	*Penicillium janthinellum*	
*Cladosporium sp.*	*Penicillium melinii*	
*Cladosporium uredinicola*	*Penicillium olsonii*	
*Emericella rugulosa*	*Penicillium oxalicum*	
*Penicillium auratiogriseum*	*Penicillium solitum*	
*Penicillium chrysogenum*	*Rhizopus stolonifer*	
*Penicillium citreonigrum*		
*Penicillium citrinum*		
*Penicillium citrium*		
*Penicillium commune*		
*Penicillium decumbens*		
*Penicillium digitatum*		
*Penicillium fellutanum*		
*Penicillium glandicola*		
*Penicillium glabrum*		
*Penicillium implicatum*		
*Penicillium italicum*		
*Penicillium melinii*		
*Penicillium miczynskii*		
*Penicillium oxalicum*		
*Penicillium paxilli*		
*Penicillium rustosum*		
*Penicillium solitum*		
*Penicillium spinulosum*		
*Penicillium variabile*		
*Penicillium waksmanii*		
*Rhizopus microsporus*		
*Rhizopus stolonifer*		

Among the most frequently encountered genera were *Aspergillus* spp. (*A. niger*, *A. flavus*, *A.japonicus*, *A.melleus*, *A.parasiticus*), *Penicillium* spp.(*P.chrysogenum*, *P.decumbens)*, *Cladosporium* sp., *Alternaria alternata*, and *Rhizopus microsporus*. These species were commonly detected in air samples collected from IDC. Similarly, in ODC samples, *A. niger*, *A. candidus*, *A. flavus*, *A. japonicus*, *Cladosporium* sp. and *Penicillium decumbens* were the most frequently identified species.

Among the most frequently encountered genera were *Aspergillus* spp. (*A. niger*, *A. flavus*, *A. japonicus*, *A. melleus*, *A. parasiticus*), *Penicillium* spp. (*P. chrysogenum*, *P. decumbens*), *Cladosporium* sp., *Alternaria alternata*, and *Rhizopus microsporus*. These species were commonly detected in air samples collected from inside display cases. Similarly, in samples taken from outside the display cases, *Aspergillus niger*, *Aspergillus candidus*, *Aspergillus flavus*, *Aspergillus japonicus*, *Cladosporium* sp., and *Penicillium decumbens* were the most frequently identified species.

Enzymatic activity assays revealed the biodeterioration potential of the isolates. Among the isolates that tested positive for cellulase activity, *A. sydowii*, *P. citreonigrum*, *P. rustosum*, *P. miczynskii*, *Rhizopus microsporus*, *Penicillium paxilli*, *Aspergillus melleus*, *Fusarium avenaceum*, *Cladosporium uredinicola*, *Penicillium melinii*, *Penicillium spinulosum*, and *Aspergillus candidus* exhibited high activity. For protease activity, *Penicillium citreonigrum*, *Cladosporium* sp., *Penicillium citrinum*, and *Penicillium melinii* were prominent.

Isolates with high zone-to-colony diameter ratios were considered primary threats to the structural integrity of textile fibers.

The results indicate that certain fungal species identified in the museum environment pose serious biological threats to artifacts not only due to their presence but also because of their high enzymatic activity. Therefore, it is concluded that indoor air quality in museums should be regularly monitored, microclimatic conditions optimized, and biological load periodically assessed.

## 4. Discussion

This study addressed the identification of microfungi commonly found on textile artifacts and within the museum environment of the Ege University Ethnography Museum, along with their enzymatic activities. The findings clearly demonstrate that museum environments pose a significant biological risk and that conservation strategies should not be limited to physical environmental conditions alone.

The CFU values determined in DG-18 and PCA media (120–450 CFU/m³ and 300–1000 CFU/m³, respectively) indicate an increased biological load particularly in poorly ventilated areas such as storage zones and IDC (inside display cases). This finding is consistent with previous studies that highlight seasonal and spatial differences in spore concentrations [12,13]. This situation suggests the presence of a persistent bioaerosol source due to inadequate microclimatic control.

The fact that most isolates with high cellulase and protease activity belonged to the genera *Aspergillus* and *Penicillium* highlights their potential to degrade both cellulosic and protein-based textile fibers. This is consistent with the fiber-degrading capabilities of these fungi reported in previous studies [5]. In particular, the cellulase activity of species such as *A. sydowii* and *P. citrinum* may significantly weaken the mechanical integrity of artifacts. Additionally, proteolytic species like *A. flavus* and *P. chrysogenum* have been shown to cause aesthetic damage such as pigment production, odor, and staining [2,4].

This study underscores the need for an integrated approach to biological risk management in museum environments. Specifically, the following strategies play a critical role in the long-term preservation of textile artifacts:

- Maintaining microclimatic conditions, particularly relative humidity within the range of 50 ± 5% and enhancing air circulation,
- Continuous monitoring of air quality and microbial load,
- Regular biological analyses and surface/environmental disinfection,
- Targeted interventions against species with high enzymatic activity potential.

## 5. Conclusion and recommendations

This study comprehensively assessed the risk of biological deterioration in textile artifacts of cultural heritage by investigating microfungal contamination within the museum environment. Air and surface samples collected from the Ege University Ethnography Museum revealed high microbial loads, a significant portion of which consisted of microfungi capable of producing destructive enzymes such as cellulase and protease. The phenotypic identification of genera such as *Aspergillus*, *Penicillium*, *Alternaria*, and *Cladosporium* confirmed the presence of widespread and potentially hazardous microbial agents in the museum setting.

Enzymatic activity assays demonstrated that these microfungi possess a high potential to degrade the fibrous structure of artifacts. The findings indicate that microbiological conservation strategies should not be limited to preventive measures alone but must also include scientific protocols for the regular monitoring of biological threats. In light of these findings, the following recommendations are proposed to enhance the protection of textile artifacts in museum environments:

1. Regular microbiological monitoring of indoor air quality in the museum,
2. Periodic assessment of microbial contamination on artifact surfaces,
3. Routine inspection of potential infection sources such as air vents and storage corners,
4. Optimization of environmental conditions (relative humidity, temperature, ventilation) instead of relying on biological agents to suppress the growth of highly enzymatic species,
5. Integration of biological risk assessment protocols into cultural heritage conservation units,
6. Training museum staff on biological risks and protection strategies,
7. Collaboration with expert microbiologists when necessary.

The results of this study provide important insights and guidance not only for the Ege University Ethnography Museum but also for other museums with similar structural and exhibition conditions.

## Supporting information

S1 Data Raw microbiological air sampling data (CFU/m^3^) obtained from DG-18 and PCA media during September and May sampling periods. (XLSX)
